# Accumulation of Arsenic in Spongy Bone

**Published:** 1890-01-11

**Authors:** 


### ACCUMULATION OF ARSENIC IN SPONGY BONE.

It is, or ought to be, well known, as we were reminded during the Maybrick trial, that even when fatal consequences have followed the prolonged injection of small doses of arsenic, none may be found in the viscera if its employment have been discontinued for a few days. Drs. Rabuteau and Dragendorf had already proved that in such cases it may to a certain extent take the place of the phosphorus in the bones; but M. Pouchet has recently shown that under such circumstances it tends to accumulate, especially in the more spongy bones, as those of the cranium and the vertebræ, where it may be detected as late as eight or ten weeks after it had ceased to be taken, and long after every trace has vanished even from the liver. This fact of its slow elimination from the bones is of the greatest value from a medico-legal point of view.

